# Line-Field Confocal Optical Coherence Tomography as a Follow-Up Tool for 3D-Printed HDR Surface Brachytherapy of Cutaneous Squamous Cell Carcinoma: A Proof-of-Concept Study at Short-Term (Six-Month) Follow-Up

**DOI:** 10.3390/cancers18182922

**Published:** 2026-09-09

**Authors:** Piotr Sobolewski, Mateusz Koper, Michal Poltorak, Pawel Banatkiewicz, Malgorzata Kolos, Lukasz Poltorak, Maciej Szwast, Irena Walecka

**Affiliations:** 1 The National Medical Institute of the Ministry of the Interior and Administration, Woloska 137, 02-507 Warsaw, Poland; 2 Chair and Clinic of Dermatology and Pediatric Dermatology, Centre of Postgraduate Medical Education, 02-507 Warsaw, Poland; 3 Electrochemistry at Soft Interfaces Team, Department of Inorganic and Analytical Chemistry, Faculty of Chemistry, University of Lodz, Tamka 12, 91-403 Lodz, Poland; 4 Department of Chemical and Process Engineering, Warsaw University of Technology, Warynskiego 1, 00-645 Warsaw, Poland

**Keywords:** squamous cell carcinoma, LC-OCT, line-field confocal optical coherence tomography, brachytherapy, 3D printing, non-invasive imaging, treatment monitoring, non-melanoma skin cancer

## Abstract

**Background/Objectives**: Cutaneous squamous cell carcinoma (cSCC) treated with high-dose-rate (HDR) surface brachytherapy using patient-specific 3D-printed applicators lacks a validated non-invasive follow-up modality capable of resolving microscopic residual disease. Line-field confocal optical coherence tomography (LC-OCT) provides real-time, cellular resolution, three-dimensional imaging of the epidermis and superficial dermis and has been proposed as a candidate tool for post-radiotherapy monitoring. We evaluated the feasibility of LC-OCT for pre- and post-treatment imaging of cSCC and assessed whether an LC-OCT-defined remission phenotype, operationally defined a priori as the absence of all malignancy-associated LC-OCT criteria, can be documented after 3D-printed HDR surface brachytherapy. **Methods**: Eight consecutive patients with histologically confirmed cSCC, unsuitable for surgery, were treated with HDR surface brachytherapy delivered through patient-specific 3D-printed applicators. LC-OCT imaging was performed immediately before brachytherapy and at short-term follow-up (six months). Nineteen pre-treatment LC-OCT criteria derived from the Cinotti 2021 descriptive criterion set for cSCC were scored: seventeen malignancy-associated criteria, plus elastosis (scored as a background photodamage covariate, not counted towards remission) and fibrosis (scored post-treatment only). Presence/absence of each criterion was rated by two blinded dermatologists; inter-assessor agreement was quantified using percentage agreement and Cohen’s kappa. **Results**: Baseline LC-OCT showed a rich malignant signature: hyperkeratosis, parakeratosis, disarranged epidermal architecture, dyskeratotic keratinocytes, atypical and crowded nuclei, and dermoepidermal junction alterations were present in eight out of eight lesions (100%) under an inclusive two-reader rule. At short-term follow-up, six months after the final brachytherapy fraction, all seventeen malignancy-associated criteria constituting the pre-specified remission set were absent in all eight patients under the decision rule described in the article—which classifies isolated dilated linear vessels occurring in an otherwise criterion-negative, fibrotic dermis as post-radiation telangiectasia rather than residual tumour, yielding an LC-OCT-defined remission phenotype rate of 100% (eight out of eight; 95% exact binomial [Clopper–Pearson] CI 63.1–100%). Post-radiation fibrosis was observed in 8/8 patients (100%), and dilated linear vessels were observed in six out of eight patients (75%, evaluated under the same decision rule (Observer 1: 4/8; Observer 2: 6/8). Elastosis, a marker of background actinic photodamage present in eight out of eight lesions at baseline and excluded from the malignancy-associated criterion count, is distinct from post-treatment fibrosis, a radiation-induced dermal remodelling feature scored only at follow-up; the two should not be conflated. **Conclusions**: In this proof-of-concept cohort, LC-OCT was feasible for pre- and post-treatment imaging of cSCC after 3D-printed HDR surface brachytherapy, produced highly reproducible dual-observer readings (κ = 0.903), and documented, at a single short-term time point six months after the final fraction, an LC-OCT-defined remission phenotype in every treated lesion, with a consistent fibrosis signature. Persistent dilated linear vessels in a fibrotic, otherwise criterion-negative dermis were classified as a non-specific post-radiation vascular change and did not preclude LC-OCT-defined complete remission. Because this remission phenotype rests on a pre-specified but not externally validated imaging definition and was not anchored to post-treatment histopathology or reflectance confocal microscopy, it constitutes an imaging surrogate rather than a confirmed complete remission, and microscopic residual disease below the resolution limit of LC-OCT cannot be excluded. These hypothesis-generating findings support LC-OCT as a candidate non-invasive monitoring modality, requiring confirmation in larger, biopsy-anchored prospective studies with longer follow-up.

## Simple Summary

Squamous cell carcinoma of the skin is normally removed surgically, but some patients cannot undergo an operation because of advanced age, coexisting illness, or the position of the tumour on the face. For them, brachytherapy is an alternative: a radiation source is brought close to the tumour through a mould shaped to the individual patient’s anatomy, in this case produced by 3D printing. Once the skin has healed, however, clinicians have no dependable way of checking whether tumour cells persist beneath the surface, because taking another biopsy from a healed field is usually neither practical nor desirable. We assessed line-field confocal optical coherence tomography, a handheld technique that images living skin at a level of detail approaching that of a microscope slide without cutting it. Eight patients with biopsy-proven tumours were imaged before treatment and again six months later. Two dermatologists, unaware of which images came from which visit, looked for an established list of features that signal this cancer. Before treatment, those features were abundant. At six months, none were visible in any patient, leaving only scarring and slightly widened vessels, both expected after radiation. The readers agreed almost completely. This small, short study cannot replace biopsy, but it supports further testing of the method.

## 1. Introduction

Cutaneous squamous cell carcinoma (cSCC) is the second most common non-melanoma skin cancer (NMSC) worldwide, and its incidence continues to rise steeply with age, cumulative ultraviolet exposure, immunosenescence, and chronic immunosuppression [1,2]. Although surgical excision with margin control remains the standard of care [3], a substantial proportion of patients are unsuitable candidates because of advanced age, multimorbidity, anticoagulation, functional or cosmetic constraints imposed by lesions on the central face, or personal preference [4,5]. For such patients, radiotherapy—particularly high-dose-rate (HDR) surface brachytherapy—offers a non-invasive, organ- and tissue-sparing alternative with demonstrated local control [6,7,8].

Standard flat applicators, however, fit poorly on the curved, mobile, and cosmetically sensitive anatomy where cSCC frequently arises, producing air gaps, dose heterogeneity, and potential underdosage of tumour margins [9,10]. Patient-specific 3D-printed applicators derived from computed tomography (CT) datasets have emerged as a means of restoring dosimetric conformity while preserving the workflow advantages of surface brachytherapy [11,12,13]. In a companion prospective study, our group recently reported the feasibility, dosimetric reproducibility, and one-year outcomes of 3D-printed HDR surface brachytherapy in a geriatric BCC cohort, using dermoscopy for post-treatment monitoring [14].

That companion study explicitly identified a limitation shared by essentially all published brachytherapy outcome literature for keratinocyte cancers: post-treatment surveillance relies on clinical inspection and, at best, dermoscopy, neither of which can resolve residual disease at cellular scale. Dermoscopy captures surface pigmentary and vascular patterns but does not visualise deeper tissue layers or cytologic atypia, so subtle residual tumour or early recurrence may go undetected until a lesion becomes clinically apparent [14,15]. In the BCC paper, we therefore proposed reflectance confocal microscopy (RCM) and line-field confocal optical coherence tomography (LC-OCT) as candidate modalities for future post-radiotherapy monitoring [14].

LC-OCT is a real-time, contactless, three-dimensional imaging technology that combines the cellular resolution of confocal microscopy with the depth penetration of conventional optical coherence tomography, producing simultaneous vertical and horizontal images of the epidermis and superficial dermis at histological resolution [16,17]. In keratinocyte carcinomas, LC-OCT has been shown to visualise architectural disarray, dyskeratotic and atypical keratinocytes, dermoepidermal junction (DEJ) alterations, broad infiltrative strands, keratin pearls, and dilated linear/glomerular vessels, and to outperform dermoscopy in specificity for the diagnosis of skin cancer [17,18,19,20]. In the seminal 2021 study by Cinotti and colleagues, nineteen LC-OCT criteria for actinic keratosis (AK) and cSCC were defined and validated against histopathology and remain the reference criterion set for cSCC imaging today [18].

While the diagnostic performance of LC-OCT in cSCC is now well established, its use as a follow-up tool after radiotherapy remains almost unexplored. Preliminary experience with topical field treatments and cryotherapy for AK has shown that LC-OCT can document the normalisation of hyperkeratosis, acanthosis, and keratinocytic atypia after successful treatment [21,22], but analogous data for cSCC after brachytherapy—a setting in which histological re-biopsy of an apparently healed field is both impractical and undesirable—are absent from the literature.

Two features of 3D-printed HDR surface brachytherapy for cSCC make a sensitive imaging-based follow-up especially attractive. First, patients selected for this modality are typically those in whom re-biopsy of the treated field is difficult or contraindicated. Second, in the present cohort, the available follow-up was short (six months), and short follow-up in a curative-intent treatment setting increases the value of any imaging modality capable of documenting a cellular-level treatment response rather than merely surface healing. In this context, we designed a proof-of-concept study to answer a narrow, tractable question: can LC-OCT document the disappearance of the cSCC criteria (Cinotti 2021) after 3D-printed HDR surface brachytherapy, and can two blinded assessors reach reproducible readings on the same images? To our knowledge, this is the first application of LC-OCT to the post-treatment monitoring of cSCC treated with patient-specific 3D-printed HDR surface brachytherapy.

## 2. Materials and Methods

### 2.1. Study Design and Ethics

This was a prospective, single-centre, single-arm proof-of-concept study of patients with histologically confirmed cutaneous SCC treated with patient-specific 3D-printed HDR surface brachytherapy at the National Medical Institute of the Ministry of the Interior and Administration in Warsaw, Poland. The study was designed as a companion investigation to our previously published BCC cohort [14], with the methodological framework (patient selection, applicator fabrication, brachytherapy delivery, blinded dual-assessor scoring) preserved and adapted for cSCC and for LC-OCT rather than dermoscopy as the imaging modality. The study was conducted in accordance with the ethical principles of the Declaration of Helsinki, and the protocol was reviewed and approved by the Ethics Committee of the Centre of Postgraduate Medical Education, Warsaw, Poland (approval number 119/2023, granted in 2023). Written informed consent to participate in the study and for the use of clinical and imaging data for publication was obtained from every patient prior to enrolment [14].

### 2.2. Patient Selection and Diagnosis

Consecutive patients with clinically suspected cSCC who were candidates for HDR surface brachytherapy were screened. Inclusion required: (i) histopathological confirmation of cSCC on punch biopsy prior to treatment, with hematoxylin–eosin staining assessed by an experienced dermatopathologist according to the WHO 2023 skin tumour guidelines; (ii) contraindication to, or refusal of, surgical excision, based on lesion location on cosmetically sensitive facial anatomy, patient comorbidity, or patient preference; and (iii) ability to tolerate CT-based planning and repeated brachytherapy sessions with a custom applicator. Patients with clinically or radiologically evident regional or distant metastasis were excluded, in line with standard indications for surface brachytherapy of cSCC [7,8].

A total of eight patients meeting these criteria were enrolled and completed treatment and post-treatment LC-OCT follow-up. Baseline demographics and lesion characteristics are summarised in Table 1.

### 2.3. 3D-Printed Applicator Fabrication

Patient-specific applicators were fabricated using the workflow validated in our BCC companion study [14,23]. In brief, CT scans of the cSCC-affected anatomical site were converted to .stl models and printed in polylactic acid (PLA, Fiberology) on fused deposition modelling machines (Prusa Mini and Prusa i3 MK3+) using a layer thickness of 0.15 mm, 100% infill, and print speeds of 25–80 mm/s depending on nozzle trajectory. Support structures were defined manually for each device to minimise surface defects while preserving mechanical reliability. Applicator geometry was verified against the original .stl model prior to first use, and repositioning stability was confirmed on repeat CT during treatment as previously described [14,23].

### 2.4. HDR Surface Brachytherapy Delivery

CT-based treatment planning was performed with the patient immobilised and the 3D-printed applicator in place, following the workflow of the BCC companion study [14]. The clinical target volume (CTV) was defined as the visible lesion with an appropriate margin; the planning target volume (PTV) accounted for positional and applicator-fit uncertainties. Dwell positions and times of the 192Ir source were optimised so that at least 90% of the PTV received the prescribed dose (D100 ≥ 90%), with dose homogeneity and skin surface hotspots evaluated on dose–volume histograms. The prescription was 51 Gy delivered in 17 fractions, matching the schedule used in our BCC companion cohort [14]. This fractionation was chosen for consistency across the institutional 3D-printed HDR surface brachytherapy programme and lies within the range of dose–fractionation schedules reported for cSCC surface brachytherapy [7,8,10].

Acute skin reactions were graded using the Radiation Therapy Oncology Group (RTOG) skin toxicity scale, as in the BCC companion study [14]. Acute skin toxicity, graded weekly during treatment and at the first post-treatment visit using the RTOG skin toxicity scale, is summarised as follows: Grade 1 in *n* = 6, Grade 2 in *n* = 2, and no Grade 3 or 4. No patient required treatment interruption or discontinuation because of acute toxicity.

### 2.5. LC-OCT Imaging Protocol

LC-OCT imaging was performed on each patient at two time points: (t1) immediately before the first brachytherapy fraction, on the same day as clinical and dermoscopic assessment; and (t2) at short-term follow-up after completion of brachytherapy (6 months from the last fraction). All acquisitions were performed with the deepLive™ LC-OCT system (DAMAE Medical, Paris, France), using the same device and imaging workflow as in our BCC companion study [14].

For every lesion, at least three representative video acquisitions covering the clinical tumour bed and its perilesional field were obtained, combining vertical (cross-sectional) and horizontal (en-face) modes to a maximum depth of approximately 500 µm and a lateral field of view of approximately 1.2 × 0.5 mm per acquisition. At follow-up, the LC-OCT probe was positioned on and around the previously treated field, with clinical photographs and dermoscopic images acquired in parallel to ensure that the imaged area corresponded to the original tumour bed and its immediate periphery.

Imaging sites at baseline were selected to maximise coverage of clinically and dermoscopically abnormal tissue: for each lesion, the treating dermatologist marked the visible tumour margin and a standardised set of 3–5 anatomical landmarks… before the first LC-OCT acquisition, and these landmarks, together with the baseline clinical and dermoscopic photographs, were used to relocate the same field at the 6-month follow-up. Because the LC-OCT lateral field of view (approximately 1.2 × 0.5 mm per acquisition) is substantially smaller than the treated area, at least three acquisitions per time point were obtained across the marked field and its periphery in a systematic raster pattern to reduce sampling error; however, exact pixel-level co-registration between the baseline and follow-up acquisitions was not possible, and residual disease confined to unimaged sub-regions of the treated field cannot be formally excluded.

### 2.6. LC-OCT Criteria and Scoring

The pre-treatment criterion set comprised the nineteen LC-OCT criteria for cSCC described by Cinotti and colleagues [18]: hyperkeratosis (stratum corneum thickness > 20 µm); parakeratosis (small dark areas corresponding to cell nuclei within the stratum corneum); erosion/ulceration (dark, sharply outlined, and irregularly contoured areas filled with amorphous material and cellular debris); acanthosis (epidermal thickness > 60 µm); disarranged epidermal architecture (variation in the shape, size, and reflectivity of keratinocyte nuclei with loss of the normally layered epidermal architecture); dyskeratotic keratinocytes (large, hyper-reflective, round intra-epidermal cells); atypical nuclei (irregular in shape and size); crowded nuclei (high nuclear density); mottled pigmentation of keratinocytes (clustered hyper-reflective keratinocytes); pigmentation of the basal layer (a hyper-reflective basal layer); keratin pearls (whorled hyper-reflective keratin accumulations); whorled appearance (whorled arrangement of intra-epidermal keratinocyte nuclei); alterations of the dermoepidermal junction (loss or interruption of the normally well-defined hyporeflective DEJ band); adnexal involvement (enlarged, atypical hair follicle epithelium); tumour budding (rounded projections of a completely disarranged epidermis composed of atypical keratinocytes with blurred outlines); broad strands (invasive strands of atypical and dyskeratotic keratinocytes interrupting the DEJ and projecting into the dermis); dilated linear vessels (well-defined hyporeflective elongated dermal areas showing intraluminal blood cell flow on LC-OCT video); glomerular vessels (hyporeflective, loosely packed, spiral-shaped superficial dermal vessels); and elastosis (hyporeflective areas in the dermis reflecting actinic damage) [18]. Of these nineteen criteria, seventeen are malignancy-associated; elastosis is scored as a background covariate of photodamage rather than a marker of malignancy, and fibrosis (below) is a post-treatment-only, radiation-related criterion. Both are therefore excluded from the seventeen-criterion malignancy set used to define the remission phenotype (Section 2.7). In the post-brachytherapy setting, isolated dilated linear vessels without any other malignancy-associated LC-OCT criteria were a priori classified as a non-specific vascular finding compatible with post-radiation telangiectasia and dermal remodelling, rather than as residual tumour-associated angiogenesis.

At post-treatment imaging, one additional criterion was scored: fibrosis, defined a priori as the presence of hyper-reflective cord-like structures within the dermis, indicative of post-radiation dermal remodelling. Fibrosis was not scored at pre-treatment because it is not a feature of untreated cSCC.

For every patient, at each time point and for each criterion, two dermatologists experienced in LC-OCT and dermoscopy (Observer 1 and Observer 2) independently scored the criterion as present (1) or absent (0) on the acquired vertical and horizontal LC-OCT videos and still frames. Both observers were blinded to (i) each other’s scores; (ii) the identity of the patient; and (iii) whether the images had been acquired before or after brachytherapy—the image order was randomised prior to review, in line with the blinding procedure of the BCC companion study [14]. Scoring was performed on the acquired image datasets alone, without access to clinical, dermoscopic, or histopathological information.

### 2.7. Definition of the LC-OCT-Defined Remission Phenotype

**Figure 1:**
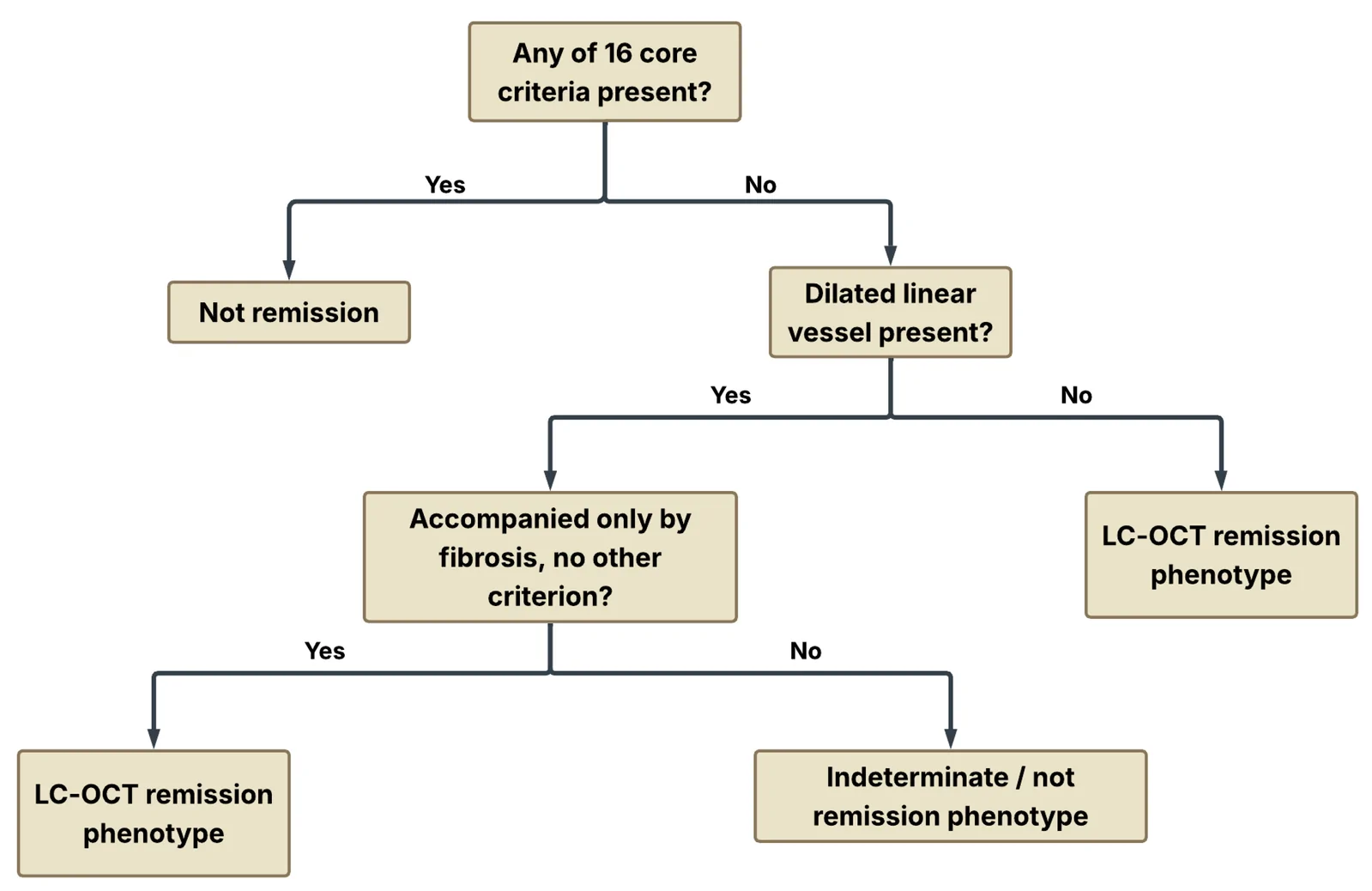
Two-step algorithm for post-treatment assessment of LC-OCT findings and classification of the LC-OCT-defined remission phenotype. **Step 1:** assessment of 16 malignancy-associated LC-OCT criteria, excluding elastosis (a background photodamage feature) and dilated linear vessels. **Step 2:** contextual assessment of dilated linear vessels. When dilated linear vessels are isolated and occur in an otherwise criterion-negative, fibrotic treated dermis, they are classified as post-radiation telangiectasia/dermal remodelling and do not preclude the LC-OCT-defined remission phenotype. When they occur with one or more of the 16 other malignancy-associated criteria, they contribute to a non-remission classification.

An a priori operational definition of the LC-OCT-defined remission phenotype (RP) was adopted for this proof-of-concept study: LC-OCT-defined remission phenotype (LC-OCT RP) was declared when all seventeen malignancy-associated LC-OCT criteria (i.e., the nineteen cSCC criteria, Cinotti 2021, excluding elastosis, which reflects background photodamage rather than tumour) were scored as absent (0) at the post-treatment time point by both blinded observers, or by at least one observer with the other scoring 0 for the remaining criteria on the same lesion. Isolated post-treatment findings of fibrosis or of persistent dilated linear vessels within an elastotic, treated dermis were, by definition, not counted towards the LC-OCT remission criterion set and were interpreted as a post-brachytherapy scarring and fibrosis phenotype (post-radiation telangiectasia and dermal remodelling), rather than as evidence of residual tumour. This definition was fixed before data extraction and is consistent with the imaging response framework used by our group in the BCC companion study [14] and with published LC-OCT criteria for the diagnosis of cSCC [18,20]. The pre-specified algorithm used to assess post-treatment LC-OCT criteria and classify the LC-OCT-defined remission phenotype is presented in Figure 1.

### 2.8. Statistical Analysis

Given the proof-of-concept nature of the study and the small sample size (*n* = 8), analyses were descriptive and hypothesis-generating. For each criterion, the number of patients scored as positive at each time point was reported separately for Observer 1, Observer 2, and using an inclusive rule (positive if either observer scored 1). Inter-assessor agreement was evaluated for binary ratings, coded as absent (0) or present (1), with each criterion–patient–time point combination constituting one paired rating. The pre-treatment analysis included 152 paired ratings (8 patients × 19 criteria), whereas the post-treatment analysis included 160 paired ratings (8 patients × 20 criteria, including post-treatment fibrosis). The pooled analysis therefore comprised 312 paired ratings. For each dataset, a 2 × 2 Observer 1-by-Observer 2 contingency table, raw percentage agreement, and Cohen’s kappa were calculated. Individual ratings and the corresponding contingency tables are provided in Supplementary Tables S1 and S2. Interpretation of kappa followed Landis and Koch: 0.61–0.80 substantial and 0.81–1.00 almost perfect agreement [24]. Given the exploratory nature of the study, the small sample size, sparse discordant pairs, and the multiplicity arising from criterion-specific comparisons, no formal inferential tests were performed for individual LC-OCT criteria. All computations were performed in Python 3.11 (NumPy 1.26) on the anonymised binary dataset extracted from the study workbook.

To enable independent verification of the reported Cohen’s κ, the complete binary rating dataset—Observer 1 and Observer 2 scores for every criterion, patient, and time point, together with the resulting 2 × 2 inter-assessor contingency table used to compute κ—is provided as Supplementary Table S1 (CSV format).

## 3. Results

### 3.1. Patients and Treatment

Eight patients with histologically confirmed cSCC were treated with 3D-printed HDR surface brachytherapy (51 Gy in 17 fractions) and completed LC-OCT imaging before and after treatment. All eight completed the prescribed brachytherapy schedule with no treatment interruptions attributable to acute toxicity. Baseline demographics and lesion sites are summarised in Table 1: six of eight patients were women, and two were men; ages ranged from 45 to 96 years (median 82 years). Lesion sites were predominantly on the face (five cheeks, one temple, one ear) with one lesion on the right foot.

### 3.2. Pre-Treatment LC-OCT Findings

The Cinotti 2021 cSCC criteria were highly prevalent on baseline LC-OCT (Table 2). Hyperkeratosis, parakeratosis, disarranged epidermal architecture, dyskeratotic keratinocytes, atypical nuclei, crowded nuclei, and DEJ alterations were observed in eight of eight patients according to both observers. Erosion/ulceration was present in seven of eight lesions according to Observer 1 and eight of eight according to Observer 2, giving an inclusive frequency of eight of eight; acanthosis was present in eight of eight (Observer 1) and seven of eight (Observer 2). Mottled pigmentation of keratinocytes was present in eight of eight patients according to Observer 1 and six of eight according to Observer 2. Whorled appearance and tumour budding were present in seven of eight lesions; broad strands in seven to eight of eight. Vascular features were more heterogeneous: dilated linear vessels in four to five of eight lesions and glomerular vessels in three to five of eight. Pigmentation of the basal layer and adnexal involvement were the least frequent malignancy-associated features (one to two of eight and two to three of eight, respectively). Elastosis, reflecting background actinic damage rather than tumour, was universally present (eight of eight).

Per patient, the number of pre-treatment malignancy-associated criteria scored as present by at least one observer ranged from 13 to 17 of 17 possible criteria (the mean was 14.5 across the entire cohort), consistent with an image dataset of unambiguously malignant, architecturally disorganised lesions with strong cytologic atypia and DEJ compromise (Table 2).

### 3.3. Post-Treatment LC-OCT Findings

At short-term follow-up (six months), both observers scored sixteen of the seventeen malignancy-associated LC-OCT criteria as absent in all eight patients (Table 2). The seventeenth criterion, dilated linear vessels, persisted in up to six of eight patients but, under the a priori decision rule (Section 2.7), was reclassified as non-specific post-radiation telangiectasia rather than residual malignancy when it occurred in isolation within an otherwise criterion-negative, fibrotic dermis; this reclassification—not the raw absence of the criterion—is what yields the 17/17 remission phenotype result reported below (Table 2). Specifically, hyperkeratosis, parakeratosis, erosion/ulceration, acanthosis, disarranged epidermal architecture, dyskeratotic keratinocytes, atypical nuclei, crowded nuclei, mottled pigmentation of keratinocytes, pigmentation of the basal layer, keratin pearls, whorled appearance, DEJ alterations, adnexal involvement, tumour budding, broad strands, and glomerular vessels all resolved in eight of eight patients. Elastosis, which had been treated by the same field radiation, was no longer identifiable as a distinct dermal feature at follow-up in any patient in the treated field, likely reflecting post-radiation remodelling of the dermis rather than a bona fide resolution of long-standing photodamage. Individual patient-level pre-treatment criterion counts, post-treatment residual malignancy-associated findings, additional post-radiation features, and the resulting LC-OCT-defined remission phenotype classification are summarised in Table 3.

Two features persisted or newly appeared at follow-up. Dilated linear vessels—a non-specific feature that overlaps with post-radiation telangiectasia—were present in four of eight patients according to Observer 1 and six of eight according to Observer 2, giving an inclusive prevalence of six of eight (75%). Post-treatment fibrosis, defined as the presence of hyper-reflective cord-like structures within the dermis, was observed in eight of eight patients by both observers. In line with the a priori remission definition, such isolated post-treatment dilated linear vessels in a fibrotic, otherwise criterion-negative dermis were interpreted as post-radiation telangiectasia and scarring and did not contribute to the count of residual malignancy-associated LC-OCT criteria.

Under the a priori operational definition in Section 2.7, all eight patients achieved the LC-OCT-defined remission phenotype. No lesion showed residual dyskeratotic keratinocytes, atypical nuclei, broad strands, or DEJ interruption on either observer’s reading; this reflects the absence of an LC-OCT imaging signature of malignancy rather than histopathologically confirmed tumour clearance, since no post-treatment biopsy was obtained (see Discussion, Limitations). No clinical or LC-OCT recurrences were observed during the available follow-up (six months).

### 3.4. Inter-Assessor Agreement

**Figure 2:**
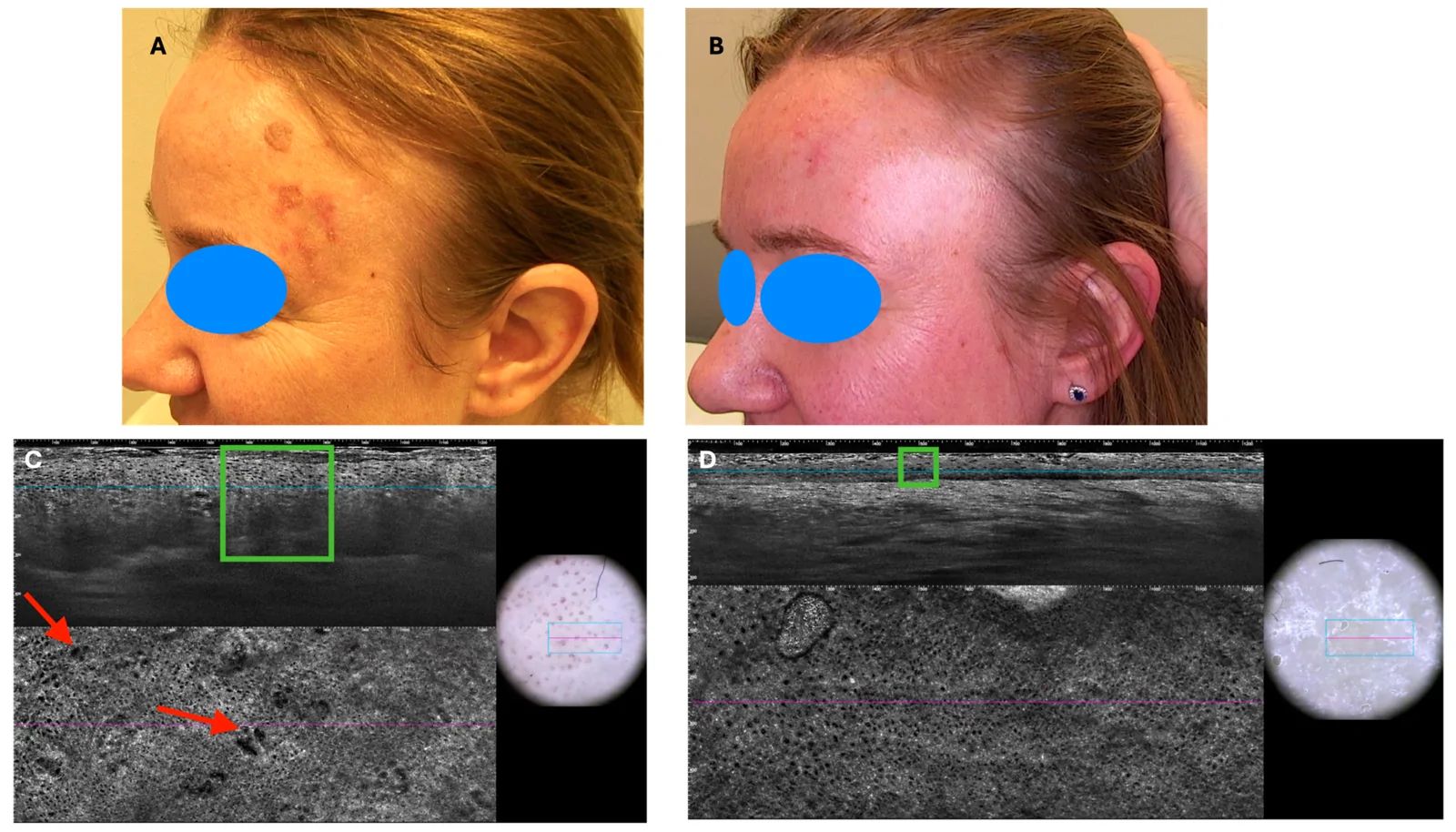
(**A**,**B**) Clinical pictures of patient No. 2 with SCC of the left temple ((**A**)—pre-treatment, (**B**)—post-treatment, 6-month follow-up). (**C**,**D**) LC-OCT pictures at the time of (**A**,**B**) pictures were taken; the most visible characteristics are indicated: red arrows–glomerular vessels, green squares–the difference in the epidermal thickness before and after treatment.

**Figure 3:**
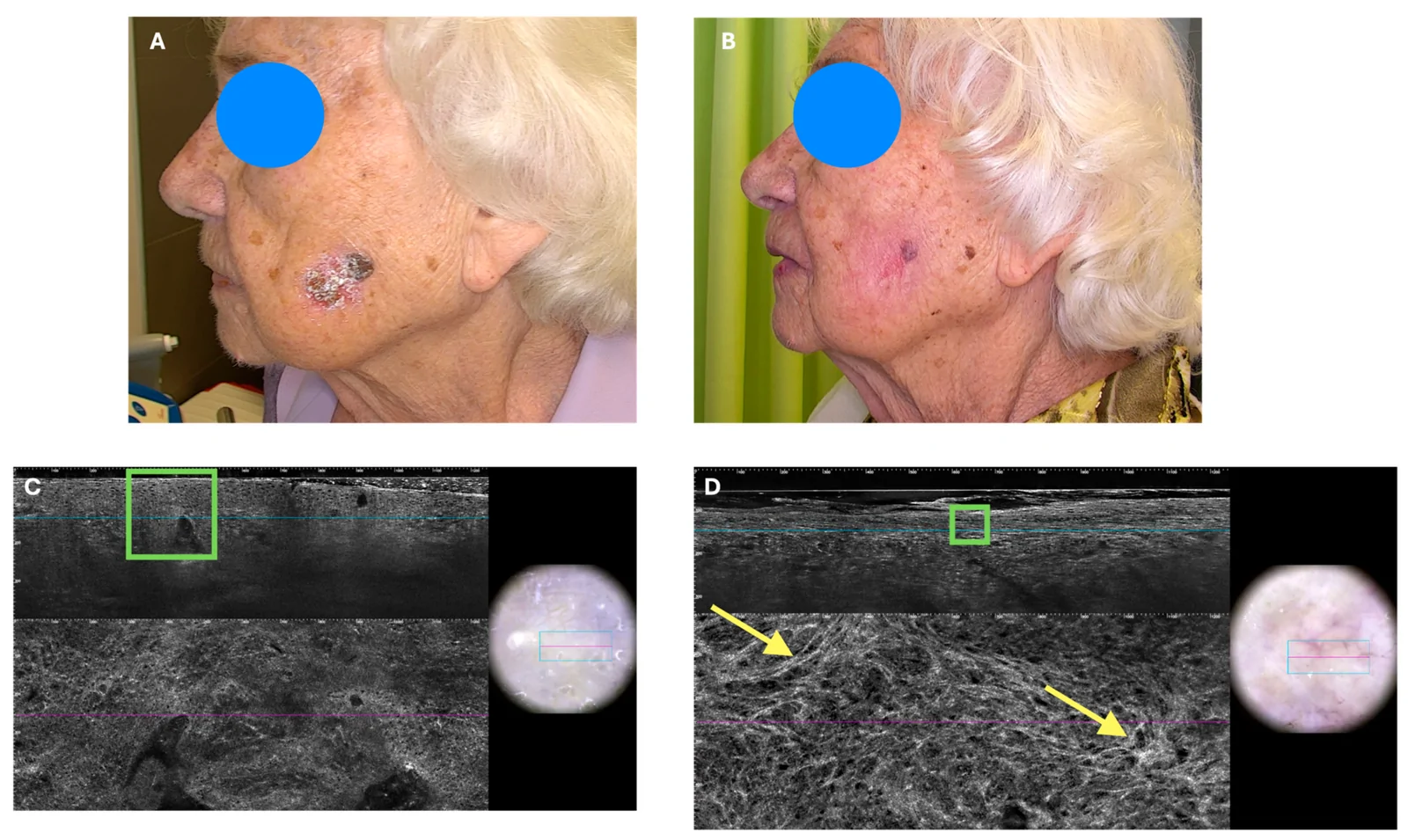
(**A**,**B**) Clinical pictures of patient No. 4 with SCC of the left cheek ((**A**)—pre-treatment, (**B**)—post-treatment 6-month follow-up). (**C**,**D**) LC-OCT pictures at the time of (**A**,**B**) pictures were taken; the most visible characteristics are indicated: green squares—the difference in the epidermal thickness before and after treatment, yellow arrows—fibrosis in the DEJ after treatment.

Across all 312 criteria (8 patients × [19 pre-treatment + 20 post-treatment criteria]), overall agreement between the two observers was 95.2%, corresponding to Cohen’s κ = 0.903 (almost perfect agreement) [24]. Agreement was substantial at baseline (91.4%, κ = 0.712, *n* = 152 pairs), where richer, denser image content and criterion-level ambiguity (e.g., mottled pigmentation, keratin pearls, broad strands vs. tumour budding) produced most of the discordance, and almost perfect after treatment (98.8%, κ = 0.916, *n* = 160 pairs), where the vast majority of ratings were concordantly negative. These figures are comparable to the almost-perfect inter-rater reliability (κ = 0.879) reported by our group for dermoscopy in the BCC companion cohort [14]. Criterion-specific changes were therefore reported descriptively (Figure 2 and Figure 3).

## 4. Discussion

In this proof-of-concept study of eight patients with cutaneous squamous cell carcinoma treated with patient-specific 3D-printed HDR surface brachytherapy, LC-OCT documented a rich, cellular-level pre-treatment signature consistent with the diagnostic criteria of Cinotti and colleagues, and a uniform disappearance of sixteen of the seventeen malignancy-associated criteria at short-term follow-up, with the seventeenth (dilated linear vessels) reclassified as post-radiation telangiectasia under the pre-specified decision rule. All eight patients therefore fulfilled the a priori definition of the LC-OCT-defined remission phenotype. Inter-assessor agreement was substantial to almost perfect (κ = 0.712 pre-treatment, κ = 0.916 post-treatment, κ = 0.903 overall), demonstrating that the Cinotti 2021 cSCC criteria can be applied reproducibly by two blinded readers to both untreated and irradiated skin in this specific clinical context. To our knowledge, this is the first application of LC-OCT to the post-treatment monitoring of cSCC after 3D-printed HDR surface brachytherapy.

These findings extend the companion dermoscopy study in three ways. First, the imaging modality itself resolves features that dermoscopy cannot: dyskeratotic keratinocytes, atypical nuclei, crowded nuclei, DEJ interruption, and broad strands are all defined at cellular scale and require a depth-resolved cellular imaging tool such as LC-OCT or RCM [16,17,18,19,20]. Second, whereas in the BCC dermoscopy study certain features (telangiectasia, white shiny clods, MAY globules) persisted after brachytherapy and were interpretable only as post-treatment inflammatory or reparative changes, the LC-OCT signature after brachytherapy in the present cSCC cohort was strikingly clean. Only two features remained: post-radiation fibrosis (in eight of eight patients) and dilated linear vessels (in up to six of eight patients)–both of which are established components of the post-radiation skin phenotype and are interpreted, under our pre-specified decision rule, as markers of tissue remodelling rather than residual tumour; this interpretation has not been verified against histopathology in the present cohort. Consistent with this interpretation, persistent dilated linear vessels in the absence of any other malignancy-associated LC-OCT criteria were considered part of the post-radiation telangiectatic and fibrotic response and were not used to downgrade the LC-OCT-defined remission phenotype. Third, the strong post-treatment concordance between observers (κ = 0.916) suggests that, at least for lesions in which brachytherapy has been successful, LC-OCT criteria are easier to score after treatment than before it, because the untreated cSCC image is architecturally noisier than the fibrotic, atrophic post-radiation dermis.

The prevalence of pre-treatment features observed in our cohort is consistent with the descriptive literature. Cinotti and colleagues, in a series of 108 cSCC lesions, reported hyperkeratosis, parakeratosis, disarranged epidermal architecture, dyskeratotic keratinocytes, atypical nuclei, crowded nuclei, and DEJ interruption among the most frequent SCC criteria, with broad strands and interrupted DEJ characterising invasive tumours [25]. Ruini and colleagues similarly identified epidermal disruption, keratinocyte atypia, dilated/glomerular vessels, and interrupted DEJ as recurrent LC-OCT features of keratinocyte carcinomas [19]. A subsequent head-to-head comparison of LC-OCT and RCM showed that LC-OCT was superior to RCM for the visualisation of parakeratosis, dyskeratotic keratinocytes, and dilated/glomerular vessels, suggesting that LC-OCT may be the more informative modality for cSCC surveillance where both are available [26].

The universality of post-treatment fibrosis (eight of eight) is expected and clinically informative. Hyper-reflective, cord-like dermal fibrosis is the LC-OCT correlate of the well-recognised post-radiation dermal remodelling seen histologically and on ultrasound after HDR brachytherapy [27]. Its consistent detection in every treated patient in our cohort supports the biological plausibility of the LC-OCT signal in this specific treatment context. Persistent dilated linear vessels (up to six of eight) can be reasonably interpreted as post-radiation telangiectasia—a pattern paralleling the persistence of telangiectasia observed dermoscopically after brachytherapy in the BCC companion cohort—rather than as residual tumour-associated angiogenesis [28]. Importantly, the specific tumour-associated vascular pattern of glomerular vessels, which was present in up to five of eight patients before treatment, resolved completely (zero of eight) in all patients after treatment, supporting a genuinely regressive interpretation of the vascular findings.

Two limitations of the dermoscopy-based follow-up used in the BCC companion study are directly addressed by LC-OCT. First, dermoscopy could not resolve DEJ status or cytological atypia; LC-OCT can. Second, dermoscopy relied on inclusive *p*-value patterns that cannot, on their own, exclude microscopic residual disease. LC-OCT, while itself not histopathology, provides a substantially higher-resolution surrogate: the demonstrated pooled sensitivity and specificity of LC-OCT for keratinocyte skin cancers are 87% and 91%, respectively, and diagnostic confidence with LC-OCT has been shown to improve by nearly 25% over dermoscopy alone [19,29,30]. Our observation that sixteen of seventeen malignancy-associated criteria were absent in every treated patient, with the seventeenth (dilated linear vessels) reclassified as post-radiation telangiectasia, is evidence—at the resolution of a non-invasive cellular imaging technique, not of histopathology—of the absence of an LC-OCT imaging phenotype of residual tumour. This is not equivalent to histopathological exclusion of residual disease and should not be interpreted as such.

At the same time, this study has several important limitations. The sample was small (*n* = 8), and the treatment intent was curative, so the population was inherently enriched for good responders and biased against the detection of local recurrence. Follow-up was short (six months), which precludes any conclusion about medium- or long-term local control; late recurrences of cSCC after radiotherapy are documented in the literature, and the true test of LC-OCT as a follow-up modality is whether it detects such recurrences earlier than clinical inspection or dermoscopy. No post-treatment histopathological or RCM correlate of the remission phenotype was available, so microscopic residual disease below the resolution limit of LC-OCT—including disease beyond its ~500 µm imaging depth (see below)—cannot be excluded, a limitation shared and acknowledged in the dermoscopy-based BCC companion study. In addition, the a priori operational definition of the LC-OCT remission phenotype adopted here has not been externally validated. This operational definition also assumes that isolated post-treatment dilated linear vessels in a fibrotic dermis represent post-radiation telangiectasia and scarring rather than low-volume residual tumour-associated angiogenesis, an assumption that will need to be tested in larger, biopsy-anchored cohorts. Cohen’s kappa estimates should be interpreted descriptively. Multiple criterion-level ratings were nested within only eight patients, and post-treatment ratings were dominated by concordant negative findings; consequently, the reported values should not be regarded as precise or generalisable estimates of inter-reader reliability. A formal generalisability study involving multiple centres and readers is therefore needed. The study was also single-centre and single-arm, without a control comparator such as dermoscopy alone or clinical inspection alone, so the incremental clinical benefit of LC-OCT over dermoscopy in this treatment setting remains to be quantified prospectively.

In addition, because the LC-OCT field of view is small relative to the treated area, follow-up imaging relied on anatomical landmarking rather than pixel-level co-registration with baseline images, so a degree of sampling error in relocating the identical tumour bed cannot be excluded.

A further, clinically important constraint is the limited imaging depth of LC-OCT, approximately 500 µm, which restricts visualisation to the epidermis and superficial (papillary) dermis. Because this cohort did not systematically record baseline tumour thickness or Breslow-equivalent level of dermal invasion, and because cSCC selected for brachytherapy can extend into the reticular dermis or along peri-adnexal structures well beyond this depth, the absence of superficial malignancy-associated criteria on post-treatment LC-OCT cannot exclude residual disease confined to deeper dermal planes. This depth limitation is a more fundamental constraint on the inference than the absence of histopathological confirmation alone, and future studies should pair LC-OCT with a depth-capable modality (e.g., high-frequency ultrasound or MRI) or with targeted biopsy in lesions with documented deep invasion at baseline.

A confirmatory study should therefore be prospective, multicentre, and adequately powered; enrol a larger, biopsy-anchored cSCC cohort, ideally with a mix of in situ and invasive disease; follow patients for at least 12–24 months with structured LC-OCT, dermoscopy, and clinical assessments; and correlate any suspicious post-treatment LC-OCT finding with a targeted biopsy. The inclusion of RCM as a within-patient comparator would help to define the incremental value of LC-OCT specifically. Blinded, independent, multi-reader scoring—including readers external to the treatment team—would test the generalisability of the Cinotti 2021 criteria to irradiated skin. Finally, the reproducibility framework used here, namely blinded dual-assessor binary scoring, Cohen’s κ, and an explicit operational definition of the LC-OCT remission phenotype, is directly transferable to such a study.

## 5. Conclusions

In this proof-of-concept study of eight patients with cutaneous squamous cell carcinoma treated with patient-specific 3D-printed HDR surface brachytherapy, LC-OCT was feasible for pre- and post-treatment imaging, produced substantial to almost perfect inter-assessor agreement (κ = 0.903 overall), and documented an LC-OCT-defined remission phenotype in every treated lesion, with a consistent post-radiation fibrosis signature and no evidence of residual dyskeratotic keratinocytes, atypical nuclei, DEJ interruption, or broad strands, other than post-radiation telangiectasia attributable under the pre-specified decision rule. These findings support LC-OCT as a candidate non-invasive follow-up modality for cSCC after brachytherapy and are consistent with, and extend, the dermoscopy-based response documentation reported by our group in a BCC companion cohort. The findings should be regarded as hypothesis-generating and require confirmation in larger prospective, multicentre, biopsy-anchored studies with longer follow-up before LC-OCT can be recommended for routine follow-up of cSCC after 3D-printed HDR surface brachytherapy.

## Figures and Tables

**Table 1:** Baseline demographics and lesion characteristics of the eight patients with cutaneous squamous cell carcinoma treated with patient-specific 3D-printed HDR surface brachytherapy.

Patient	Age	Sex	Tumour Localization
1	84	male	Right ear
2	45	female	Left temple
3	96	female	Left cheek
4	95	female	Left cheek
5	87	female	Right foot
6	77	female	Right cheek
7	87	male	Left cheek
8	87	female	Left cheek

**Table 2:** Frequency of LC-OCT criteria at baseline (pre-treatment) and short-term follow-up (post-treatment) in eight patients with cutaneous squamous cell carcinoma treated with 3D-printed HDR surface brachytherapy. Values are reported separately for Observer 1, Observer 2, and using an inclusive rule (positive if either observer scored 1). Fibrosis was defined a priori as a post-treatment-specific criterion; elastosis is a background photodamage feature and is shown for completeness.

Criterion	Pre-Treatment (*n* = 8)			Post-Treatment (*n* = 8)		
	Obs 1	Obs 2	Either	Obs 1	Obs 2	Either
Hyperkeratosis	8/8	8/8	8/8	0/8	0/8	0/8
Parakeratosis	8/8	8/8	8/8	0/8	0/8	0/8
Erosion/ulceration	7/8	8/8	8/8	0/8	0/8	0/8
Acanthosis	8/8	7/8	8/8	0/8	0/8	0/8
Disarranged epidermal architecture	8/8	8/8	8/8	0/8	0/8	0/8
Dyskeratotic keratinocytes	8/8	8/8	8/8	0/8	0/8	0/8
Atypical nuclei	8/8	8/8	8/8	0/8	0/8	0/8
Crowded nuclei	8/8	8/8	8/8	0/8	0/8	0/8
Mottled pigmentation of keratinocytes	8/8	6/8	8/8	0/8	0/8	0/8
Pigmentation of the basal layer	1/8	2/8	2/8	0/8	0/8	0/8
Keratin pearls	4/8	5/8	6/8 *	0/8	0/8	0/8
Whorled appearance	7/8	7/8	7/8	0/8	0/8	0/8
Dermoepidermal junction alterations	8/8	8/8	8/8	0/8	0/8	0/8
Adnexal involvement	2/8	3/8	3/8	0/8	0/8	0/8
Tumour budding	7/8	7/8	7/8	0/8	0/8	0/8
Broad strands	7/8	8/8	8/8	0/8	0/8	0/8
Dilated linear vessels	4/8	5/8	5/8	4/8	6/8	6/8
Glomerular vessels	3/8	5/8	5/8	0/8	0/8	0/8
Elastosis (background)	8/8	8/8	8/8	0/8	0/8	0/8
Fibrosis (post-treatment only)	-	-	-	8/8	8/8	8/8

* Inclusive rule: a patient was counted as positive if either observer assigned a score of 1. For keratin pearls, the positive ratings comprised three patients identified by both observers, one identified only by Observer 1, and two identified only by Observer 2, giving 6/8 unique positive patients.

**Table 3:** Per-patient LC-OCT outcomes. Pre-treatment count refers to the number of seventeen malignancy-associated Cinotti 2021 criteria (excluding elastosis, a background-photodamage covariate, and fibrosis, a post-treatment-only criterion) scored as present by at least one observer. ‘LC-OCT remission phenotype’ (renamed from ‘LC-OCT CR’) indicates fulfillment of the decision rule in Section 2.7.

Patient	Pre-Treatment SCC Criteria Present (Either Observer)	Post-Treatment Residual SCC Criteria	Post-Treatment Additional Features	LC-OCT-Defined Remission Phenotype
1	14/17 malignancy-associated criteria	None	Fibrosis; dilated linear vessel (Obs 2 only)	**Yes**
2	15/17 malignancy-associated criteria	None	Fibrosis; dilated linear vessels	**Yes**
3	17/17 malignancy-associated criteria	None	Fibrosis	**Yes**
4	17/17 malignancy-associated criteria	None	Fibrosis	**Yes**
5	15/17 malignancy-associated criteria	None	Fibrosis; dilated linear vessels	**Yes**
6	15/17 malignancy-associated criteria	None	Fibrosis; dilated linear vessel (Obs 2 only)	**Yes**
7	15/17 malignancy-associated criteria	None	Fibrosis; dilated linear vessels	**Yes**
8	15/17 malignancy-associated criteria	None	Fibrosis; dilated linear vessels	**Yes**

## Data Availability

Data are contained within the article or Supplementary Material.

## Supplementary Materials

The following supporting information can be downloaded at: https://www.mdpi.com/article/10.3390/cancers18182922/s1. Table S1: Individual Observer 1 and Observer 2 binary ratings (present/absent) for all 17 malignancy-associated criteria, elastosis, and fibrosis, across all 8 patients and both time points; Table S2: inter-assessor agreement analysis; Representative de-identified LC-OCT image datasets for Patients 2 and 4, corresponding to Figure 2 and Figure 3, are available at: https://zenodo.org/records/22060631 (accessed on 22 August 2026).

## Abbreviations

The following abbreviations are used in this manuscript:

BCC	Basal cell carcinoma
cSCC	Cutaneous squamous cell carcinoma
LC-OCT RP	LC-OCT-defined remission phenotype
CT	Computed tomography
CTV	Clinical target volume
DEJ	Dermoepidermal junction
HDR	High-dose-rate
LC-OCT	Line-field confocal optical coherence tomography
NMSC	Non-melanoma skin cancer
PLA	Polylactic acid
PTV	Planning target volume
RCM	Reflectance confocal microscopy
RTOG	Radiation Therapy Oncology Group
